# The potential role of GLP-1 receptor agonists in substance use disorders – a systematic review

**DOI:** 10.3389/fphar.2025.1702448

**Published:** 2026-01-02

**Authors:** K. M. Völker, B. L. H. Prechtl, N. L. Bormann, D. S. Choi

**Affiliations:** 1 Department of Molecular Pharmacology and Experimental Therapeutics, Mayo Clinic College of Medicine, Rochester, MN, United States; 2 Paracelsus Medical University, Nuremberg, Germany; 3 Department of Cardiology, Klinikum Nürnberg, Nuremberg, Germany; 4 Department of Psychiatry and Psychology, Mayo Clinic College of Medicine, Rochester, MN, United States

**Keywords:** addiction, alcohol use disorder, GLP-1 recepter agonist, neuropharmacology, substance use disorder, systematic review, translational medicine

## Abstract

**Background:**

Substance use disorders (SUDs) remain a major global health challenge with limited pharmacological treatment options and high relapse rates. Glucagon-like peptide-1 receptor agonists (GLP-1RAs), originally developed for type 2 diabetes and obesity, have recently gained attention for their potential effects on addictive behaviors. Preclinical and early clinical studies suggest that GLP-1RAs modulate mesolimbic dopaminergic signaling and attenuate reward-driven behaviors, positioning them as promising candidates for novel interventions in addiction medicine.

**Methods:**

We conducted a systematic literature review in accordance with PRISMA 2020 guidelines, with protocol registration in PROSPERO (CRD42024571356). Searches were performed across PubMed, Embase, Web of Science, PsycINFO, and the Cochrane Library up to January 2025. Eligible studies included preclinical and clinical investigations examining GLP-1RAs in alcohol, nicotine, cocaine, and opioid use disorders. Data were extracted on study design, sample characteristics, interventions, and primary outcomes related to substance use behavior and neurobiological mechanisms.

**Results:**

A total of 41 studies were included, comprising 35 preclinical and six clinical investigations. Preclinical evidence consistently demonstrated that GLP-1RAs, including exendin-4, liraglutide, and semaglutide, reduced substance intake, relapse-like behaviors, and cue-induced drug seeking across multiple drug classes. These effects were linked to GLP-1R activation in brain reward circuits, including the nucleus accumbens, ventral tegmental area, and nucleus of the solitary tract. Clinical studies provided preliminary support, particularly for alcohol use disorder, with GLP-1RAs showing reductions in alcohol consumption and craving in several clinical trials. However, clinical findings remain heterogeneous and limited by small sample sizes and short study durations.

**Conclusion:**

This systematic review highlights GLP-1RAs as a promising therapeutic approach for SUDs, targeting the neurobiological pathways underlying both metabolic and reward regulation. While preclinical data are convincing, clinical evidence remains preliminary, underscoring the need for larger, well-designed randomized controlled trials. GLP-1RAs may represent a novel pharmacological strategy bridging metabolic and neuropsychiatric domains in addiction treatment.

**Systematic Review Registration:**

https://www.crd.york.ac.uk/PROSPERO/view/CRD42024571356.

## 1 Introduction

Substance use disorders (SUDs) are a major public health burden, characterized by high relapse rates and limited treatment efficacy. The complex interaction of neurobiology and psychosocial factors has hindered the development of targeted pharmacotherapies. Glucagon-like peptide-1 receptor agonists (GLP-1RAs), originally developed for type 2 diabetes, have recently emerged as promising candidates for addiction treatment (Klausen et al., 2022). Acting on GLP-1 receptors both peripherally and in central reward-related regions such as the nucleus accumbens (NAc) and ventral tegmental area (VTA), GLP-1RAs modulate dopaminergic signaling and may reduce the reinforcing effects of addictive substances, offering a novel therapeutic approach (Klausen et al., 2022; Probst et al., 2023). Clinically, GLP-1RAs constitute a class of synthetic analogues of the endogenous incretin hormone GLP-1, which is derived from proglucagon and secreted by enteroendocrine L-cells in response to nutrient ingestion (Inkretin-Mimetika, 2022). These agents include exenatide, liraglutide, dulaglutide, semaglutide, lixisenatide, and albiglutide, which differ in molecular structure, half-life, and dosing frequency—from short-acting twice-daily peptides to long-acting acylated analogs suitable for weekly or oral administration. Beyond their glucose-lowering and appetite-suppressing effects, GLP-1RAs exert anti-inflammatory and neuroprotective actions mediated via GLP-1 receptors in the intestinal immune system and central nervous system. These pleiotropic mechanisms may extend their therapeutic potential beyond metabolic disorders to neuropsychiatric and addiction-related conditions (Drucker, 2018). An overview of currently approved GLP-1RAs and their pharmacological properties is provided in Supplementary Material 1.

Notably, early preclinical work had already demonstrated that central GLP-1 receptor activation influences reward-related behavior beyond metabolic regulation. In particular, activation of GLP-1 receptors in the VTA was found to decrease the reinforcing efficacy of cocaine, while stimulation of GLP-1 signaling within the NAc and laterodorsal tegmental nucleus reduced alcohol consumption and reward sensitivity (Schmidt et al., 2016; Vallöf et al., 2016). These foundational findings established GLP-1 as a key modulator of mesolimbic reward circuitry and provided the conceptual basis for its later exploration in addiction. SUDs share a common neurobiological basis characterized by dysregulated dopaminergic signaling within the mesolimbic reward system, particularly projections from the VTA to the NAc that mediate reinforcement across diverse addictive substances (Tuesta et al., 2017). It could be demonstrated that nicotine activates GLP-1–expressing neurons in the nucleus tractus solitarius (NTS) projecting to the medial habenula–interpeduncular nucleus pathway, where GLP-1 receptor activation suppresses drug reward by enhancing excitatory drive in the IPN and promoting avoidance (Tuesta et al., 2017). Another study further showed that systemic administration of the GLP-1RA liraglutide attenuated nicotine self-administration, reinstatement, and withdrawal-induced hyperphagia in rodents, while highlighting convergent GLP-1-mediated modulation of dopaminergic transmission in reward-related regions also implicated in alcohol and other SUDs (Herman et al., 2023). Together, these findings support a mechanistic model in which GLP-1 receptor activation counteracts mesolimbic dopamine dysregulation, a shared substrate underlying addiction and relapse (Tuesta et al., 2017; Herman et al., 2023).

Preclinical research provides support for the efficacy of GLP-1RAs in reducing substance-seeking behaviors and relapse rates across various SUDs. In alcohol use disorder, GLP-1RAs such as semaglutide and liraglutide have been shown to attenuate alcohol-induced dopamine release in the NAc and reduce relapse-like drinking behavior (Aranäs et al., 2023b; Vallöf et al., 2016) Furthermore, the effects of GLP-1RAs extend to other substances, including nicotine and opioids. For example, liraglutide has been shown to decrease nicotine-seeking behaviors in rodent models, while exenatide reduced drug-induced reinstatement of heroin-seeking behaviors (Herman et al., 2023; Douton et al., 2021). However, findings regarding the efficacy of GLP-1RAs in cocaine addiction have been less consistent compared to other substances. Several studies suggest that the therapeutic effects of GLP-1RAs in this context may be contingent upon distinct neurobiological mechanisms within the mesolimbic reward circuitry, particularly involving dopaminergic signaling in the mesolimbic reward system (Hernandez et al., 2019; Schmidt et al., 2016).

Despite these advances, several critical questions remain unanswered. First, the mechanisms underlying the differential efficacy of GLP-1RAs across substances are not fully understood. Factors such as sex differences, baseline levels of substance use, and co-occurring metabolic conditions appear to influence treatment outcomes, yet the precise nature of these interactions requires further investigation (Díaz-Megido and Thomsen, 2023; Vallöf et al., 2020). Second, while preclinical evidence highlights the potential of GLP-1RAs in reducing relapse and substance-seeking behaviors, clinical studies are limited in number, often characterized by small sample sizes and short follow-up durations. For instance, it was reported that exenatide reduced alcohol cue reactivity in individuals with alcohol use disorder, particularly in those with a high body mass index, but these findings are not yet generalizable to broader populations (Klausen et al., 2022). Similarly, another clinical trial found that dulaglutide reduced alcohol consumption during smoking cessation, though its effects were less pronounced in heavy drinkers (Probst et al., 2023).

Another key question pertains to the long-term safety and efficacy of GLP-1RAs in the treatment of SUDs. While short-term trials suggest that these agents are well-tolerated, the potential for sustained efficacy in treating chronic SUD remains unclear (Vallöf et al., 2020). Additionally, the integration of GLP-1RAs into existing therapeutic frameworks—whether as monotherapies or in combination with established treatments—has yet to be thoroughly explored. Preclinical findings suggest that GLP-1RAs may exhibit synergistic effects when paired with other pharmacological agents targeting addiction-related pathways, such as ghrelin antagonists (Abtahi et al., 2018). However, these hypotheses require rigorous testing in clinical trials.

The aim of this review is to systematically evaluate the therapeutic potential of GLP-1RAs in treating SUDs, with a focus on their mechanisms of action, variability in efficacy across substances and populations, and potential for clinical application. This work will integrate findings from both preclinical and clinical studies to address three central objectives. First, to elucidate the neurobiological mechanisms by which GLP-1RAs modulate addiction-related pathways. Second, to identify factors influencing variability in treatment outcomes, including sex differences and baseline substance use, and third, to assess the emerging clinical applicability of GLP-1RAs for SUDs, based on findings from available clinical trials, and to discuss their potential integration into current therapeutic frameworks, considering preclinical evidence and existing treatment modalities.

## 2 Methods

### 2.1 Methodological framework, study design and protocol registration

This systematic review was conducted in accordance with the (PRISMA, 2020) statement. The study protocol was prospectively registered with the International Prospective Register of Systematic Reviews. (PROSPERO; registration number: CRD42024571356). (PROSPERO, 2021) A complete (PRISMA 2020) Checklist is provided in Supplementary Material 2 to ensure methodological transparency (PRISMA, 2020).

### 2.2 Search strategy

A comprehensive search was developed and deployed on 8 August 2024, by a medical librarian in consultation with the study investigators. This search was conducted across five major databases: PubMed, Scopus, APA PsycINFO, Embase, and the Cochrane Central Register of Controlled Trials. The literature search was limited to studies published within the last 10 years to ensure temporal relevance, methodological consistency, and scientific currency. This timeframe was chosen to capture the most recent preclinical and clinical developments in the field. To broaden coverage, the reference lists of all included articles and key systematic reviews were manually screened for additional eligible studies. Importantly, the search targeted both clinical and preclinical studies, facilitating a thorough, translational assessment of the evidence base. A comprehensive list of search queries and strategies is provided in Supplementary Material 3.

### 2.3 Eligibility criteria

Eligible studies included randomized controlled trials (RCTs), clinical trials with control groups, and preclinical animal studies. Clinical trials with control groups were included to extend the scope of controlled evidence, while preclinical animal studies were deemed essential for investigating the mechanisms by which GLP-1RAs influence substance use behavior. The study population included human participants aged 18 years and older, diagnosed with a SUD according to the Diagnostic and Statistical Manual of Mental Disorders, Fifth Edition (DSM-V) criteria (APA, American Psychiatry Association, 2013). Preclinical studies using animal models specifically designed to investigate substance use were also included, ensuring clinical relevance and translational potential. The intervention of interest was limited to GLP-1RAs receptor agonists, such as semaglutide, liraglutide, exenatide, dulaglutide, and lixisenatide. Only peer-reviewed articles published in the past 10 years were considered to ensure the review incorporated the most current and scientifically rigorous evidence. Exclusion criteria included observational studies without control groups, case reports, expert opinions, reviews, and non-peer-reviewed articles (e.g., commentaries and letters to the editor).

### 2.4 Study selection and screening process

The search yielded 2869 records, which were exported into Zotero for reference management and subsequently uploaded into Covidence, a systematic review software designed to streamline evidence synthesis workflows. In addition to manual sorting, Covidence was used to automatically detect and remove duplicates, resulting in a final dataset of 1,544 unique records subjected to formal screening. Two independent reviewers (KV and BP) screened all 1,544 titles and abstracts using predefined eligibility criteria. To prevent erroneous exclusions, any study where disagreement arose was passed to the next screening phase for full-text review. During full-text review, two reviewers (NB and KV) independently evaluated the full texts of 214 studies for eligibility. Disagreements were resolved through independent re-evaluation and consensus discussions among the authors, with DSC having the final say. Ultimately, 41 studies met all inclusion criteria and were incorporated into the final systematic review. Reasons for exclusion at the full-text stage were tracked, most frequently due to wrong study types (51), speaker abstracts (47), wrong study designs (32), wrong outcomes (11) and other reasons (11).

**FIGURE 1:**
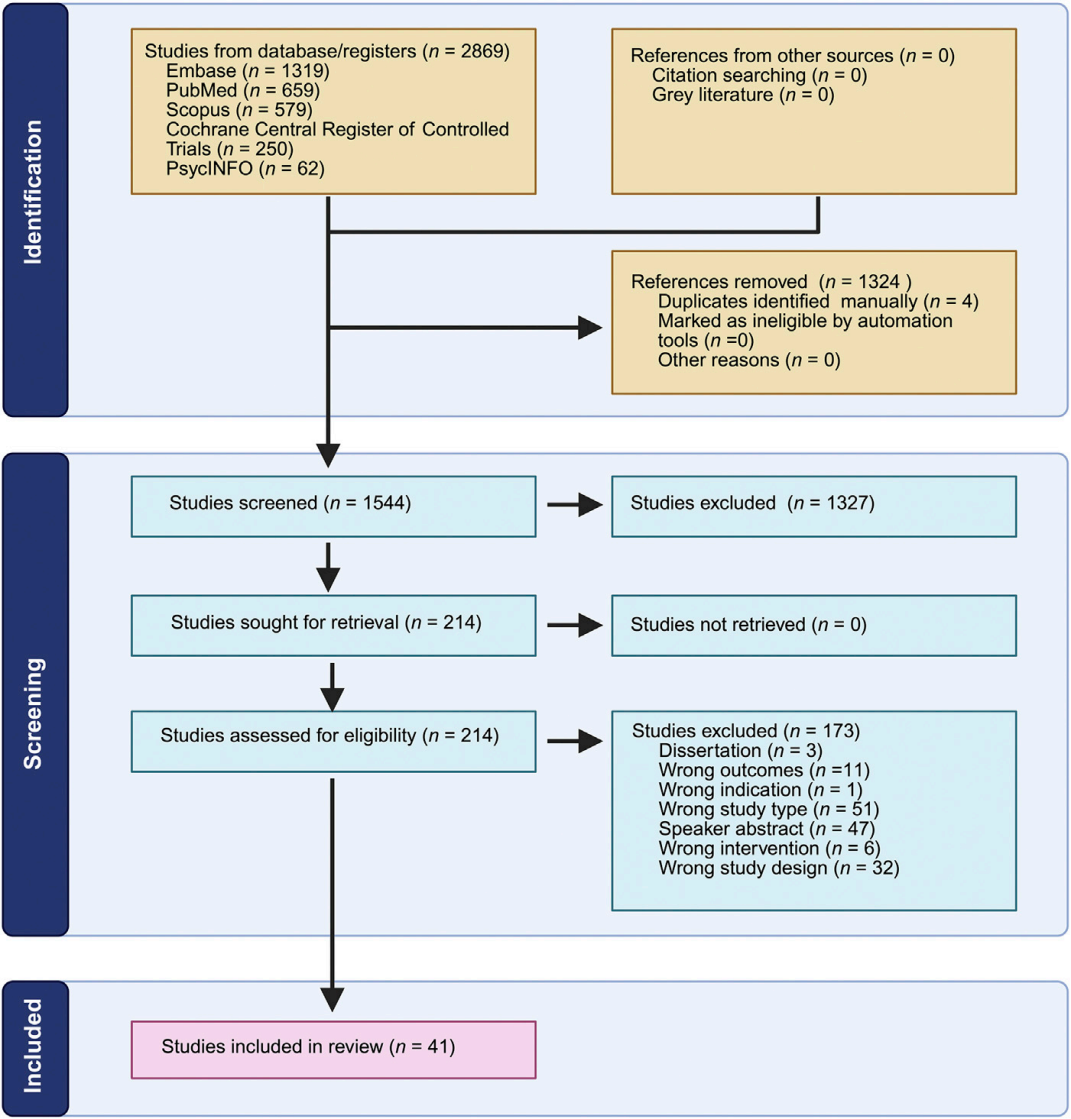
Flowchart illustrating the study selection process for a review. Identification shows 2,869 studies from various sources with 1,324 references removed. Screening includes 1,544 studies, excluding 1,327. Of the 214 assessed for eligibility, 173 are excluded for reasons such as incorrect outcomes and study type. Finally, 41 studies are included in the final review. Figure was created with BioRender.com.

To visually represent the study identification and selection process, a (PRISMA 2020) flow diagram (PRISMA, 2020) was constructed. It is depicted below as Figure 1. This standardized flowchart transparently details each stage—from initial database search through duplicate removal, screening, and full-text assessment—culminating in the final count of included studies. Following completion of the systematic search, one additional relevant clinical study (Hendershot et al., 2025) meeting all inclusion criteria was identified through manual tracking and was subsequently included, resulting in a total of 42 studies included in the review.

### 2.5 Resolution of disagreements

Any disagreements regarding study eligibility were resolved through discussion. If consensus could not be reached, a third reviewer (DSC) was consulted to make the final decision.

### 2.6 Recording decisions

All decisions related to study inclusion were documented in Covidence, which facilitated tracking of the selection process, management of references, and recording of reasons for exclusion during the full-text screening stage.

### 2.7 Data extraction

For each eligible study, data were extracted independently using pre-designed tables. Extracted data included general study characteristics (e.g., first author, journal, year, type of study, substance investigated, study duration), participant details (e.g., age and gender distribution in treatment and control groups for clinical studies; species, strain, and gender for preclinical studies), intervention specifics (type and dosage of GLP-1 receptor agonist and substance), comparator information, outcomes and statistical methods.

### 2.8 Methodology: risk of bias and certainty assessment of evidence

To ensure methodological rigor, the quality of included randomized controlled trials (RCTs) was assessed using the Cochrane Risk of Bias 2.0 (RoB 2, 2023). A revised Cochrane risk-of-bias tool for randomized trials (RoB 2 tool, n. d.) tool. This framework evaluates the randomization process, deviations from intended interventions, missing outcome data, measurement of outcomes, and selection of reported results. Each domain was rated as either “low risk,” “some concerns,” or “high risk,” and these informed an overall risk of bias judgment for each study. Assessments were performed independently by two reviewers (KV and BP), with disagreements resolved by consensus. For preclinical studies, the risk of bias was qualitatively assessed using adapted criteria that focused on randomization, blinding, reporting of outcomes, and sample size considerations. This allowed a structured appraisal of study reliability and translational value.

### 2.9 Data synthesis

Evidence from preclinical and clinical studies was synthesized narratively. Findings were grouped by substance class (alcohol, nicotine, cocaine, opioids) and study type (preclinical vs. clinical). The synthesis emphasized outcome measures, intervention characteristics, population differences, and mechanistic insights, with particular attention to translational relevancevidence was synthesized narratively due to high heterogeneity and the limited number of available clinical trials. Instead, similarities and divergences across studies were highlighted to contextualize the strength and limitations of the current evidence base.

### 2.10 Patient and public involvement

This study did not involve patient representatives or members of the public in its design, conduct, or reporting. No primary data collection was performed.

## 3 Results

The following section outlines the results of the systematic review including an overview of all included studies and a synthesis of both preclinical and clinical findings regarding the effects of GLP-1 receptor agonists on various substance use disorders.

### 3.1 Overview of included studies

A total of 42 primary studies met the eligibility criteria and were included in the present systematic review. These studies comprised clinical investigations in humans and preclinical experimental studies conducted in animal models. The included studies examined the potential role of GLP-1RAs in modulating substance use–related behaviors and outcomes across a broad range of substance classes.

Among the included publications, six studies were clinical in nature, comprising RCTs, pilot studies, and secondary analyses of behavioral or metabolic outcomes in individuals with SUDs. 36 studies were conducted in preclinical models, predominantly involving rodents (rats and mice), with a minority employing nonhuman primates (e.g., alcohol-preferring vervet monkeys). Preclinical studies employed validated behavioral paradigms such as intravenous or oral self-administration, cue- and drug-induced reinstatement, operant conditioning tasks, and conditioned place preference to investigate substance-seeking behavior. Clinical studies primarily addressed alcohol and nicotine use disorders and evaluated pharmacological effects on consumption, craving, abstinence, and associated metabolic or neuroendocrine parameters. In the included studies, alcohol was examined most frequently (24), followed by cocaine (8), nicotine (5), and opioids. Several studies addressed more than one substance class or reported multiple experimental conditions, including divergent GLP-1RA regimens or distinct behavioral outcomes within the same publication.

The GLP-1RAs investigated included exendin-4, liraglutide, semaglutide, and dulaglutide, with marked variability in dosing protocols across studies. Semaglutide and liraglutide were most frequently used in the context of alcohol and opioid use. Exendin-4 was the most studied GLP-1RA in nicotine and cocaine models. Dulaglutide was evaluated exclusively in clinical trials for smoking cessation and alcohol use. Preclinical studies involved acute and chronic administration paradigms, with treatment durations ranging from single-dose interventions to protocols lasting several weeks. Delivery methods included systemic (intraperitoneal, subcutaneous) and intracerebral (e.g., VTA, NAc) administration. Clinical trials ranged from two to 26 weeks in duration, with outcome assessments conducted during and after treatment.

A detailed overview of dosing regimens and corresponding clinical outcomes for all included human studies is presented in Table 1, which summarizes the administered GLP-1 receptor agonists, doses, treatment durations, study populations, and key behavioral outcomes observed across alcohol and nicotine use disorders. Across the included clinical studies, participants were adults diagnosed with SUDs or engaged in habitual substance use behaviors. A randomized controlled trial from 2022 investigated exenatide in patients meeting DSM-5 criteria, (Klausen et al., 2022), while a more recent laboratory-based trial published in 2025 examined semaglutide in a similar cohort (Hendershot et al., 2025).

**TABLE 1:** Summary of all clinical studies investigating the effects of GLP-1 receptor agonists (GLP-1RAs) on substance use disorders (SUDs), including alcohol, nicotine, and smoking cessation contexts. The table presents agent, dose, treatment duration, sample size, study population, and main outcomes as reported in the included trials.

Study	GLP-1RA (dose; duration)	Sample size (n)	Population/Substance	Main outcomes
Klausen et al. (2022)	Exenatide 2.0 mg weekly; 26 weeks	127	Alcohol use disorder	No overall ↓ in heavy drinking days; ↓ alcohol cue reactivity in ventral striatum; significant effect in BMI >30 kg/m^2^
Hendershot et al. (2024)	Semaglutide 0.25–1.0 mg weekly; 9 weeks	48	Alcohol use disorder	↓ alcohol self-administration in lab setting; inconsistent effect on heavy drinking days; well tolerated
Probst et al. (2023)	Dulaglutide 1.5 mg weekly; 12 weeks	255	Smoking cessation (secondary alcohol analysis)	↓ weekly alcohol intake 29%, adjusted: 36%, no extra effect in heavy drinkers
Lengsfeld et al. (2023)	Dulaglutide, 1.5 mg weekly; 12 weeks	255	Smokers (seeking cessation)	↓ post cessation weight gain, GI-symptoms common, no serious adverse events; ↓HbA1C levels
Yammine et al. (2021)	Exenatide ER 2 mg weekly; 6 weeks	84 (mITT 82)	Smokers (during cessation with NRT)	↑ abstinence rates; ↓ withdrawal symptoms; mitigated post-cessation weight gain; well tolerated

Two further investigations focused on nicotine dependence: one assessed dulaglutide during a 12-week smoking-cessation program, and another explored semaglutide in individuals with regular nicotine use who were not actively seeking cessation (Probst et al., 2023; Lengsfeld et al., 2023). In addition, a 2021 clinical trial on smokers receiving nicotine replacement therapy confirmed tobacco dependence using standardized cessation criteria (Yammine et al., 2021). None of the trials explicitly limited participation to treatment-seeking individuals, and addiction severity ranged from moderate to severe based on baseline consumption metrics and clinical interviews. Commonly used psychiatric and behavioral assessment tools included validated instruments such as the Alcohol Use Disorders Identification Test (AUDIT), the Fagerström Test for Nicotine Dependence and standardized craving and withdrawal scales. All clinical studies employed GLP-1RA doses identical to those used in approved metabolic indications—namely, exenatide 2 mg once weekly, dulaglutide 1.5 mg once weekly, and semaglutide up to 1.0 mg once weekly. None of the studies applied minimum BMI thresholds for inclusion. However, trials investigating exenatide and semaglutide in alcohol use disorder reported that participants with higher baseline body mass index showed more pronounced treatment effects (Klausen et al., 2022; Hendershot et al., 2025). Reported adverse events were consistent with the known safety profile of GLP-1RAs, primarily mild gastrointestinal symptoms such as nausea and decreased appetite. No serious treatment-related adverse events were reported in any of the included trials.

For preclinical investigations, a comprehensive list of all dosing protocols, experimental conditions, and associated outcomes is presented in Supplementary Material 4. These studies displayed substantial variability in dosage, administration routes, and measured endpoints, reflecting the heterogeneity across experimental models and substance classes.

### 3.2 Outcome measures

The included studies assessed a broad spectrum of outcomes encompassing behavioral, neurobiological, and clinical domains. Behavioral endpoints comprised measures such as substance intake, drug-seeking behavior, relapse-like reinstatement, cue- or stress-induced responding, and operant motivation. Neurobiological and metabolic outcomes included alterations in dopaminergic signaling, circulating GLP-1 and insulin levels, neuroinflammatory markers, and activation of reward-related brain regions. Clinical and subjective parameters focused on daily substance use, craving intensity, abstinence duration, weight changes, and overall treatment tolerability. While preclinical studies commonly reported reductions in drug-related behaviors under GLP-1RA treatment, clinical findings were more heterogeneous across trials. Some human studies indicated potential efficacy—particularly in alcohol and nicotine use—whereas others, including trials in cocaine use disorder, reported no significant treatment effects.

### 3.3 Systematic review

GLP-1RAs have been evaluated across diverse substance use disorders, encompassing alcohol, nicotine, cocaine, and opioid paradigms. Clinical investigations have primarily targeted alcohol and nicotine dependence, whereas preclinical studies have extended these observations to psychostimulant and opioid models, elucidating convergent modulatory effects on mesolimbic dopaminergic and reward-related circuitry.

#### 3.3.1 Alcohol use disorder

Two clinical studies have examined the effects of GLP-1RAs on AUD. A randomized, placebo-controlled trial investigated exenatide in individuals with AUD. While no overall reduction in heavy drinking days was observed, secondary analyses revealed significant reductions in participants with a body mass index (BMI) above 30 kg/m^2^. Additionally, exenatide reduced alcohol cue reactivity in the ventral striatum. (Klausen et al., 2022). In addition, another research group conducted a randomized, placebo-controlled trial with 51 individuals with AUD who received weekly semaglutide (0.25–1 mg) for 8 weeks (Hendershot et al., 2025). The primary outcome, alcohol self-administration in a controlled laboratory setting, showed reduced alcohol intake in the semaglutide group, while effects on heavy drinking days were less consistent. A secondary analysis of a randomized trial examining dulaglutide for smoking cessation also assessed its impact on alcohol consumption. Dulaglutide led to a 29% reduction in weekly alcohol intake, which increased to 36% when adjusted for education level. This effect was independent of baseline alcohol consumption, though the heaviest drinkers did not exhibit significant reductions (Probst et al., 2023).

Preclinical studies provide substantial support for the use of GLP-RAs in treating AUD. Semaglutide significantly reduced alcohol consumption and relapse-like drinking behaviors in rodent models (Aranäs et al., 2023b; Chuong et al., 2023; Fink-Jensen et al., 2024). These effects were attributed to the attenuation of alcohol-induced dopamine release in the NAc, a region central to the processing of reward. Furthermore, the study observed increased dopamine metabolism and elevated levels of dopamine metabolites (Aranäs et al., 2023a). In a 2017 mouse study, administration of exendin-4 after a period of alcohol deprivation significantly reduced relapse-like drinking behavior. Treatment delayed the onset of renewed alcohol intake and decreased the number of drinking bouts without affecting their duration or size, indicating a selective reduction in alcohol-seeking rather than general consumption. These effects were specific to alcohol, as water intake remained unchanged, suggesting targeted modulation of alcohol-related reward behavior.

In a related study, the influence of the GLP-1 RA Exendin-4 on intravenous ethanol self-administration (IVSA) in mice was examined (Sørensen et al., 2016). Pretreatment with Exendin-4 significantly reduced intravenous ethanol intake by up to 70%, indicating a potent suppressive effect of GLP-1 receptor activation on alcohol consumption. This reduction was not accompanied by a decrease in operant responses for a palatable liquid food reward, demonstrating that Exendin-4’s effects were specific to alcohol and not related to general motivational deficits or metabolic factors. Furthermore, ethanol IVSA paradigm often reached toxic levels in the absence of Exendin-4, underscoring the potency of ethanol as a reinforcer in this model. The study highlighted the role of GLP-1RA in directly modulating alcohol reinforcement (Sørensen et al., 2016). Within the ventral tegmental area (VTA), GLP-1 receptor signaling appears to play a specific role in modulating alcohol intake. In a controlled rodent experiment, local administration of exendin-4 markedly reduced alcohol self-administration in high alcohol drinkers, whereas low drinkers remained unaffected. These findings suggest that GLP-1 receptor activation may have a stronger effect in individuals with elevated baseline consumption levels. The intervention did not influence reacquisition or motivation to obtain alcohol, suggesting that VTA-mediated GLP-1 signaling primarily affects ongoing consumption rather than relapse behavior. Furthermore, food intake and locomotor activity remained unchanged, confirming that the observed reductions in alcohol intake reflected targeted modulation of reward-related mechanisms rather than nonspecific behavioral suppression (Dixon et al., 2020).

Further supporting these findings, targeted administration of Exendin-4 into reward-related brain regions in male Sprague-Dawley rats markedly reduced alcohol intake. The strongest effects were observed following injections into the VTA and NAc, whereas no significant changes occurred in the arcuate nucleus, paraventricular nucleus, or basolateral amygdala. Exendin-4 also diminished food-motivated behavior in several of the same regions but not within the basolateral amygdala, indicating that GLP-1 receptor activation selectively modulates specific neural circuits of reward. These data highlight the relevance of GLP-1R signaling in core mesolimbic structures and its potential therapeutic value for AUD (Colvin et al., 2022). Building on these findings, localized activation of GLP-1 receptors in the lateral septum, NAc, and ventral hippocampus was shown to markedly reduce alcohol-reinforced behavior in mice. The most pronounced effects occurred within the lateral septum, whereas stimulation of the caudate putamen produced no significant changes, underscoring the regional specificity of GLP-1R signaling. The observed reduction in alcohol self-administration was associated with decreased dopaminergic activity in both the NAc and lateral septum, highlighting the role of GLP-1 receptor activation in modulating mesolimbic reward circuits (Allingbjerg et al., 2023). In female rats, targeted microinjections into the NAc revealed that concurrent modulation of ghrelin and GLP-1 signaling within the shell region significantly reduced alcohol consumption. Administration of the GLP-1 receptor agonist exendin-4 (Ex-4) and the ghrelin antagonist JMV2959 produced a pronounced decrease in alcohol intake when delivered into the nucleus accumbens shell, whereas no such effect was observed in the core. The combined administration yielded an enhanced reduction than either treatment alone. Notably, Ex-4 also decreased food intake following NAcC infusion, while JMV2959 did not, underscoring a substance-specific modulation of reward processing within accumbal subregions (Abtahi et al., 2018).

Further elucidating the neural mechanisms, studies have emphasized the importance of GLP-1R activation in the NTS (Vallöf et al., 2019b). Demonstrated that Exendin-4 administration into the NTS significantly inhibited alcohol-induced locomotor stimulation in mice and reduced alcohol consumption in rats that had been consuming alcohol chronically. This reduction was dose-dependent and persisted at multiple time points (1-h, 4-h, and 24-h). Additionally, Exendin-4 in the NTS blocked alcohol-induced dopamine release in the NAc shell and impaired alcohol reward memory in a conditioned place preference paradigm. Importantly, pharmacological suppression of GLP-1 receptors in the NTS negated the effects of systemic Exendin-4, confirming the critical role of this region in mediating the observed behavioral effects (Vallöf et al., 2019b).

Chuong and colleagues further supported these findings by demonstrating the efficacy of semaglutide across both non-dependent and alcohol-dependent rodent models. However, its effects on GABAergic transmission appeared more complex in alcohol-dependent animals, likely due to neuroadaptations resulting from chronic alcohol exposure (Chuong et al., 2023). In a comparative investigation, it was observed that semaglutide produced a faster and more pronounced reduction in alcohol consumption than liraglutide. Interestingly, the concurrent administration of a GLP-1 receptor antagonist did not abolish these effects, indicating that GLP-1RAs may influence alcohol-related behaviors through both receptor-dependent and independent pathways (Marty et al., 2020). Further experimental evidence confirmed that semaglutide monotherapy effectively reduced alcohol consumption in both male and female rats. Combining semaglutide with bupropion or varenicline offered no synergistic benefit, and a high-fat diet alone was sufficient to reduce alcohol intake, with no further effect under combined conditions.

A subsequent primate study further extended these findings by examining the effects of semaglutide in alcohol-preferring male vervet monkeys. Treatment with semaglutide led to a marked reduction in alcohol intake, particularly during the first 2 weeks of administration, without affecting food or water consumption or body weight. During the washout phase, alcohol intake returned to baseline levels, suggesting that sustained exposure is required to maintain treatment efficacy. These results provide compelling preclinical evidence for semaglutide’s potential in the treatment of AUD and support its further evaluation in human trials (Fink-Jensen et al., 2024).

Another preclinical rodent study also demonstrated that liraglutide significantly reduced alcohol intake in high-alcohol-consuming rats, with repeated administration lowering both preference and consumption without affecting overall fluid intake. Moreover, liraglutide prevented the alcohol deprivation effect (ADE), where alcohol intake spikes after a period of abstinence, which is a model of relapse in human AUD treatment (Vallöf et al., 2016). These results mirror the findings of Thomsen et al. (2017), deeper emphasizing the relapse-prevention potential of GLP-1RAs.

Subsequent work expanded upon these findings by highlighting the role of GLP-1 receptor activation in reducing alcohol-induced locomotor activity and modulating alcohol reward memory (Vallöf et al., 2019a). Their work demonstrated that GLP-1 receptor activation via Exendin-4 (Ex4) significantly reduced alcohol-induced locomotor stimulation in rodent models, particularly when infused into the NAc shell and the laterodorsal tegmental area, suggesting that GLP-1R in these regions modulates the acute stimulating effects of alcohol (Vallöf et al., 2019a). Furthermore, it could be shown by the same study, that Ex4 reduced alcohol consumption in high alcohol-consuming rats when infused into the NAc shell and laterodorsal tegmental area, but not in low consumers, indicating a targeted effect on heavy drinkers. These region-specific effects were not observed when Ex4 was infused into other areas, such as the anterior ventral tegmental area. Additionally, GLP-1 receptor expression in the NAc shell was elevated in high alcohol-consuming rats, and there was a positive correlation between receptor expression and alcohol intake, suggesting that chronic alcohol consumption may upregulate GLP-1R expression and enhance responsiveness to GLP-1R agonists in individuals with higher alcohol use (Vallöf et al., 2019a).

Sex-dependent effects of GLP-1RAs were observed in a study that explored the influence of Exendin-4 on alcohol reinforcement in C57BL/6J mice (Díaz-Megido and Thomsen, 2023). Male mice exhibited significant reductions in alcohol-seeking behavior and alcohol self-administration with Exendin-4, whereas female mice did not show significant changes. This highlights a notable sex difference in the efficacy of GLP-1RAs for AUD (Díaz-Megido and Thomsen, 2023).

Another recent study provided additional clarity, demonstrating sex-specific differences in the long-term impact of GLP-1R agonists like dulaglutide (Vallöf et al., 2020). In this study, male rats exhibited a persistent reduction in ethanol intake even after treatment discontinuation, whereas female rats returned to baseline ethanol consumption shortly after treatment cessation (Vallöf et al., 2020). This suggests that males may experience more prolonged benefits from GLP-1R agonist treatments, while females may require continuous therapy for sustained effects. Additionally, GLP-1 receptor expression in the NAc shell was elevated in high-alcohol-consuming rodents and positively correlated with alcohol intake (Vallöf et al., 2020), suggesting that chronic alcohol consumption may upregulate GLP-1R expression, enhancing responsiveness to GLP-1R agonists in individuals with higher alcohol use. The study also found that dulaglutide altered neurotransmitter signaling in reward-related brain regions, further emphasizing the role of GLP-1R agonists in modulating reward pathways that underlie alcohol consumption (Vallöf et al., 2020). Moreover, they showed that the effects of Exendin-4 on alcohol consumption were dose-dependent and that reductions in alcohol intake persisted at various time points, including 1-h, 4-h, and 24-h intervals. These findings support the hypothesis that GLP-1R agonists can provide sustained modulation of alcohol consumption over time.

In a mechanistic study, the interaction between ghrelin and GLP-1 in ethanol intake was examined, particularly in combination with psychostimulants like D-amphetamine and cocaine. Ghrelin increased ethanol consumption when administered into the VTA and potentiated the effects of psychostimulants on ethanol intake, likely through ghrelin-1a receptor activation and interactions with dopamine D1/D2 receptors, thereby enhancing mesolimbic dopamine signaling (Colvin et al., 2022). In contrast, GLP-1 receptor activation via Ex-4 reduced ethanol consumption and blocked the effects of psychostimulants on ethanol intake. Ex-4 also counteracted ghrelin-enhanced ethanol consumption by suppressing dopamine signaling in the mesolimbic pathway, likely through reduced dopamine activity in the VTA and altered gene expression in the NAc (Colvin et al., 2022). These findings underline GLP-1RAs’ potential in modulating reward mechanisms and addressing co-use of alcohol and psychostimulants (Colvin et al., 2022).

Two complementary studies provided foundational insights into the role of GLP-1 receptor signaling in regulating reward-related behaviors relevant to substance use disorders (Sirohi et al., 2016; Suchankova et al., 2015). In a mouse model study, it was demonstrated that peripheral administration of Exendin-4 blocked amphetamine-induced conditioned place preference (CPP) in control (FLOX) mice, but not in GLP-1R KDNestin mice, where GLP-1Rs were removed from the central nervous system. Similarly, Exendin-4 significantly reduced alcohol intake in FLOX mice but had no effect on alcohol consumption in GLP-1R KDNestin mice, highlighting the critical role of central GLP-1R signaling in controlling alcohol consumption (Sirohi et al., 2016).

Parallel human–animal evidence further supports these findings: genetic variants of the GLP-1R gene have been linked to increased alcohol self-administration in humans, and pharmacological GLP-1R agonism has reduced alcohol consumption in alcohol-dependent mice. Notably, the effects of GLP-1R agonists, such as AC3174, were specific to alcohol without affecting food or water intake, underscoring the role of GLP-1R signaling in targeting substance-related behaviors without broadly impacting other consumptive behaviors (Suchankova et al., 2015).

In a nonhuman primate model of alcohol preference, treatment with the GLP-1 receptor agonists exenatide and liraglutide produced differential effects on alcohol intake. Exenatide led to a transient reduction in alcohol consumption during the first week following reintroduction, whereas liraglutide induced a more consistent and pronounced decrease throughout the treatment period. Animals receiving liraglutide exhibited significantly lower plasma alcohol levels, without evidence of toxicity. Neither agent affected water intake or produced adverse effects (Thomsen et al., 2019).

Complementary experiments in rodent models explored the effects of Ex-4 on reward salience evoked by neuropeptide Y (NPY) and ghrelin (Brigande et al., 2023). Both peptides increased operant responding for sucrose pellets and ethanol intake, but Ex-4 dose-dependently blocked these effects. Lower doses of Ex-4 (0.01 µg) reduced the stimulatory effects of NPY and ghrelin on food and ethanol intake without producing significant inhibitory effects on their own. Higher doses (0.1 µg) significantly inhibited reward-related behaviors (sucrose and ethanol intake) in the absence of NPY or ghrelin. Additionally, the combined effects of NPY and ghrelin, which enhance reward behavior, were effectively countered by pretreatment with Ex-4, indicating that these peptides may act through a common neural pathway within the ventral tegmental area. This finding reinforces the potential for GLP-1 receptor agonists to modulate reward pathways associated with alcohol and other substance use (Brigande et al., 2023).

#### 3.3.2 Tobacco use disorder

Clinical investigations have also explored the potential of GLP-1 receptor agonists in tobacco use disorder. In a randomized, placebo-controlled trial involving 46 smokers who were not actively seeking cessation, treatment with semaglutide (2.4 mg weekly for 12 weeks) led to measurable reductions in daily cigarette consumption, verified by self-report and carbon monoxide validation. The treatment was well-tolerated and not associated with any serious adverse events (Lengsfeld et al., 2023). Also, dulaglutide significantly reduced post-cessation weight gain, with dulaglutide-treated participants losing 1 kg compared to a 1.9 kg weight gain in the placebo group (Lengsfeld et al., 2023). These findings remained unchanged after a 12-month follow-up period of 12 months (Lüthi et al., 2024).

Similarly, a pilot study demonstrated that exenatide, combined with nicotine replacement therapy (NRT), significantly improved smoking abstinence rates, mitigated post-cessation weight gain and also reduced withdrawal symptoms (Yammine et al., 2021).

GLP-1RAs have also demonstrated potential in modulating nicotine addiction in preclinical models. For example, it could be demonstrated that liraglutide significantly reduces nicotine self-administration and relapse behavior in both male and female rats. Liraglutide not only attenuated nicotine-seeking during periods of abstinence but also diminished the reinstatement of nicotine-seeking following nicotine priming. Notably, the administration of liraglutide mitigated withdrawal-induced hyperphagia and the associated weight gain, particularly in female rats (Herman et al., 2023).

The mechanisms by which GLP-1RAs modulate nicotine addiction have been further clarified through studies on neuronal pathways. A study revealed that nicotine activates GLP-1-expressing neurons within the nucleus tractus solitarius (NTS), a region associated with satiety and avoidance signaling (Tuesta et al., 2017). Activation of GLP-1 receptors in these neurons was found to decrease nicotine intake and reduce nicotine-seeking behavior by modulating the medial habenula-interpeduncular nucleus (MHb-IPN) circuit, a key pathway involved in nicotine reward processing (Tuesta et al., 2017).

#### 3.3.3 Cocaine use disorder

A clinical investigation evaluated the effects of exenatide on cocaine self-administration in non-treatment-seeking individuals with cocaine use disorder. No significant differences were observed between exenatide and placebo groups regarding the number of cocaine infusions, self-reported euphoria, or craving. Nonetheless, treatment was associated with reduced circulating GLP-1 and insulin levels, indicating potential metabolic modulation without direct behavioral impact. The study’s small sample size and single-dose design, however, limit the generalizability of these findings (Angarita et al., 2021).

Preclinical investigations have further elucidated the role of GLP-1 receptor agonists in cocaine use disorder. In rodent models, activation of GLP-1 receptors in the NAc by exenatide markedly reduced cocaine-primed reinstatement, without affecting sucrose-seeking behavior—indicating a specific effect on drug-related motivation (Hernandez et al., 2019). Furthermore, exenatide increased glutamate release in the ventral tegmental area and enhanced the excitability of medium spiny neurons in the NAc, suggesting that GLP-1RAs modulate both presynaptic and postsynaptic mechanisms in addiction (Hernandez et al., 2018).

Subsequent rodent experiments have strengthened this mechanistic understanding. Targeted administration of exendin-4 into the laterodorsal tegmental nucleus (LDTg) markedly suppressed cocaine-seeking behavior in rats, while leaving sucrose-seeking, food intake, and body weight unaffected—confirming the behavioral specificity of GLP-1R activation. These effects were driven by GLP-1 receptors expressed on GABAergic neurons within the LDTg that project to the ventral tegmental area. Moreover, stimulation of the GLP-1R-dependent circuit extending from the nucleus tractus solitarius to the LDTg similarly reduced cocaine-seeking behavior, emphasizing the central role of brainstem–midbrain GLP-1 pathways in relapse control without influencing metabolic function. (Hernandez et al., 2021).

Experimental evidence further supports the involvement of GLP-1R signaling in cocaine-related stress and neuroinflammatory mechanisms. Treatment with exenatide attenuated cocaine self-administration and prevented stress-induced relapse, an effect mediated through modulation of corticosterone pathways (Schmidt et al., 2016). Complementary findings demonstrated that exenatide also normalized cocaine-induced neuroinflammatory responses in the nucleus accumbens by downregulating NF-κB activity, thereby potentially reducing vulnerability to relapse and neurotoxic adaptation (Zhu et al., 2022).

In a series of investigations in mice, the effects of the GLP-1 receptor agonist exendin-4 (Ex-4) on cocaine self-administration in mice were examined (Sørensen et al., 2015). Exendin-4 significantly reduced both acute and chronic cocaine self-administration in a dose-dependent manner, implying that GLP-1 receptor activation decreases the reinforcing effects of cocaine (Sørensen et al., 2015). Additionally, Ex-4 attenuated cocaine-induced hyperlocomotion, highlighting the potential of GLP-1 receptor agonists in reducing behavioral stimulation associated with cocaine use (Sørensen et al., 2015). This was further supported by a reduction in cocaine-induced increases in striatal dopamine levels and c-fos expression, indicating that Ex-4 interferes with cocaine’s ability to stimulate the brain’s reward system (Sørensen et al., 2015). Importantly, the same intervention did not alter activity induced by a dopamine D1 agonist, underscoring that GLP-1R modulation acts selectively on cocaine-related reinforcement rather than on general dopaminergic tone (Sørensen et al., 2015).

#### 3.3.4 Opioid use disorder

Several preclinical studies investigated the effects of GLP-1RA on opioid-related behaviors. For instance, in preclinical models, GLP-1 receptor activation was associated with changes in orexin-related signaling pathways within reward-related brain regions (Douton et al., 2021). This effect was linked to an increase in orexin-1 receptor expression in the nucleus accumbens, a region closely associated with reward processing.

Similarly, liraglutide has shown promise in models of opioid addiction. Further experiments using liraglutide revealed a marked suppression of heroin self-administration and drug-induced reinstatement in rats with high baseline drug intake, indicating that GLP-1 receptor engagement may be particularly effective in more severe addiction profiles (Evans et al., 2022). Extending these findings, investigations in fentanyl models showed that liraglutide attenuated both cue- and drug-induced relapse-like behavior. Remarkably, its behavioral efficacy paralleled that of buprenorphine, the standard opioid substitution therapy, yet without acting as an opioid agonist itself (Urbanik et al., 2022).

Further work on liraglutide’s role in opioid addiction revealed consistent reductions in heroin self-administration across experimental models (Douton et al., 2022b). In this study, Liraglutide-treated rats consistently self-administered less heroin and avoided escalation of intake, a key marker of addiction (Douton et al., 2022b). Additionally, liraglutide reduced drug-induced reinstatement of heroin-seeking behavior following abstinence, suggesting its potential to prevent relapse. However, it had no significant effect on cue-induced heroin-seeking when tested 1 hour post-administration (Douton et al., 2022b). Importantly, liraglutide’s effects were specific to drug-related behaviors, as it did not alter natural reward-seeking or saccharin intake. The treatment was well-tolerated, with minimal side effects, and only higher doses led to slight reductions in food intake and body weight (Douton et al., 2022b).

Additional mechanistic and pharmacokinetic analyses provided further insight into liraglutide’s anti-addictive profile. In a series of relapse paradigms, the compound markedly reduced heroin-seeking across cue-induced extinction, drug-primed reinstatement, and stress-induced relapse models (Douton et al., 2022a). Notably, following administration of the stressor yohimbine, liraglutide-treated rats exhibited substantially lower heroin-seeking compared to controls, underscoring its efficacy under stress-related relapse conditions. Pharmacokinetic assessments revealed a dose-dependent increase in plasma concentrations, with maximal behavioral effects occurring approximately 6 hours after injection—suggesting a close temporal coupling between drug exposure and therapeutic efficacy. Motor coordination remained unaffected, confirming that the observed reductions in drug-seeking were not attributable to nonspecific motor impairment (Douton et al., 2022a).

In contrast to these positive findings, Bornebusch and colleagues investigated the effects of GLP-1 receptor agonist Exendin-4 (Ex4) on opioid-related behaviors in male mice, focusing on morphine-conditioned place preference, remifentanil self-administration, and withdrawal symptoms (Bornebusch et al., 2019). This study revealed that Exendin-4 did not attenuate morphine-induced conditioned place preference, remifentanil self-administration, or opioid withdrawal. In some instances, Ex4 even increased the reinforcing effects of remifentanil, suggesting that GLP-1 receptor activation may not be an appropriate treatment for opioid use disorders (Bornebusch et al., 2019). These findings imply that different GLP-1 receptor agonists might have differential efficacy across various substance use disorders, being more effective in treating alcohol and nicotine addiction but less so for opioid addiction.

Taken together, these studies highlight the potential of liraglutide as a promising therapeutic agent for opioid use disorder, particularly due to its ability to reduce opioid-seeking behaviors across a range of relapse models. Liraglutide’s unique profile - marked by its efficacy in stress- and drug-induced relapse, preservation of natural reward-seeking, and minimal side effects - positions it as a compelling non-opioid alternative for treating OUD. However, the variable efficacy across different GLP-1RAs, such as Exendin-4, emphasizes the need for further research to understand the underlying mechanisms and identify the most effective compounds and therapeutic regimens for substance use disorders.

## 4 Discussion

This systematic review mapped the broader research landscape surrounding the application of GLP-1RAs in the treatment of SUDs. This review encompassed both clinical and preclinical studies, thereby enabling a translational assessment of GLP-1RA effectiveness across experimental models. The review process was implemented via Covidence, and followed (PRISMA, 2020) guidelines (PRISMA, 2020) and PROSPERO registration protocols (PROSPERO, 2021), ensuring methodological integrity.

Among the 42 studies identified, only six were clinical trials involving human participants. The remaining studies consisted predominantly of preclinical investigations using animal models. These studies consistently demonstrated that GLP-1RAs reduce substance-seeking behavior by modulating central reward pathways, especially the mesolimbic dopaminergic system (Tuesta et al., 2017; Herman et al., 2023). This effect was observed across substances, including alcohol, nicotine, cocaine, and opioids, suggesting broad pharmacological potential.

Preclinical data indicate that GLP-1 receptor agonists attenuate alcohol-induced dopaminergic activation within mesolimbic reward pathways, providing a neurobiological rationale for their observed behavioral effects (Aranäs et al., 2023a). In line with these mechanistic findings, clinical trials—including the work by Yammine and colleagues-have shown that GLP-1 receptor activation may also be effective in smoking cessation through mechanisms overlapping with metabolic regulation (Yammine et al., 2021). This supports the view that the therapeutic potential of GLP-1RAs extends beyond metabolic disorders to include modulation of reward-related and addictive behaviors.

Beyond mesolimbic mechanisms, GLP-1 signaling within the nucleus tractus solitarius has been shown to regulate the habenulo–interpeduncular pathway, a circuit critically involved in nicotine reward and aversion (Tuesta et al., 2017). Furthermore, interactions between GLP-1 and orexin signaling within mesolimbic reward networks appear to contribute to the modulation of opioid-related behaviors. Together, these mechanisms suggest that GLP-1 receptor agonists influence addiction not only through dopaminergic reward modulation but also via integrated brainstem–limbic pathways that link visceral, motivational, and affective control (Schmidt et al., 2016).

Collectively, this growing body of interdisciplinary evidence highlights an emerging translational focus on GLP-1 receptor agonists as potential pharmacotherapies for SUDs.

### 4.1 Strengths and translational implications

The systematic review exhibited several methodological strengths. The literature search strategy was developed in consultation with Gwen Wilson, Head of Library Services at Mayo Clinic and extended across five major scientific databases. Dual independent screening, clear documentation of exclusion criteria, and use of a structured review platform (Covidence, 2016) enhanced the transparency and reproducibility of the process.

Importantly, by integrating both mechanistic (preclinical) and applied (clinical) studies, the review enabled a multilevel synthesis of GLP-1RA effects. The convergence of evidence from distinct methodological approaches strengthened the biological plausibility of GLP-1RAs as promising pharmacotherapies in addiction medicine.

Collectively, preclinical findings demonstrate that GLP-1RAs, particularly semaglutide, liraglutide and Exendin-4, consistently reduce alcohol consumption and modulate reward pathways associated with alcohol use. The studies reviewed emphasize the mechanisms through which GLP-1RAs reduce alcohol’s rewarding properties, predominantly by modulating dopaminergic and reward-related neural pathways in regions such as the nucleus accumbens, ventral tegmental area, and lateral hypothalamus (Aranäs et al., 2023a; Sørensen et al., 2016; Dixon et al., 2020; Chuong et al., 2023; Marty et al., 2020; Colvin et al., 2020; Díaz-Megido and Thomsen, 2023; Vallöf et al., 2016; Vallöf et al., 2019a; Vallöf et al., 2019b). However, the efficacy of these agents may vary depending on factors such as sex differences, baseline alcohol consumption levels, and the differential roles of central versus peripheral GLP-1R signaling (Vallöf et al., 2019b; Vallöf et al., 2019a; Sørensen et al., 2016; Thomsen et al., 2017; Thomsen et al., 2019; Sirohi et al., 2016; Suchankova et al., 2015).

From a translational perspective, this convergence of evidence supports the potential of GLP-1RA in reducing alcohol use, particularly in individuals with higher baseline alcohol consumption or in those who may be more responsive to dopaminergic modulation. The collective body of evidence supports further investigation into the use of GLP-1RAs in clinical settings for treating AUD, with particular attention to factors such as sex differences, long-term efficacy, and the specific mechanisms through which these agents modulate both central and peripheral reward pathways (Aranäs et al., 2023a; Sørensen et al., 2016; Dixon et al., 2020; Brigande et al., 2023). Abtahi et al. findings also align with the existing literature, highlighting the nucleus accumbens shell as a critical target for GLP-1 receptor agonists and ghrelin antagonists in modulating alcohol consumption and suggesting that combination therapies targeting these pathways may offer enhanced efficacy in treating AUD (Abtahi et al., 2018).

### 4.2 Research gaps

Despite these strengths, several critical research gaps were identified. Most notably, the clinical evidence base is underdeveloped and highly unbalanced, with a primary focus on alcohol and tobacco use disorders. Substances such as opioids and stimulants, which are supported by extensive preclinical evidence, remain largely unstudied in human trials. This imbalance limits generalizability and hampers the development of substance-specific interventions.

Early clinical evidence indicates that semaglutide may reduce alcohol consumption in individuals with alcohol use disorder, based on findings from a recent randomized trial (Hendershot et al., 2025). A second clinical investigation showed that dulaglutide was also associated with reduced alcohol intake, although this effect was less pronounced among individuals with heavy drinking patterns (Probst et al., 2023). Together, these findings support the notion that GLP-1 receptor activation influences alcohol-related reward processing, in line with mechanisms identified in preclinical models.

Initial clinical observations indicate that metabolic status—particularly higher baseline BMI—may shape the therapeutic response to GLP-1 receptor agonists in alcohol use disorder. The underlying mechanisms remain unclear, and targeted clinical investigations are needed to clarify this relationship.

In general, clinical trials were typically small in scale and short in duration. For example, the trial by Probst et al. lasted only 12 weeks (Probst et al., 2023), limiting the ability to assess sustained treatment effects. Another clinical trial by Klausen and collaborators reported that benefits were more pronounced in participants with an elevated body mass index (BMI), suggesting that generalizability across patient populations may be limited (Klausen et al., 2022).

Beyond the RCTs included in this systematic review, recent large-scale observational studies have provided complementary real-world evidence. For instance, a registry-based cohort including more than 200,000 individuals with AUD reported reduced alcohol-related health events among GLP-1RA users. Although such studies were not part of our predefined inclusion criteria, they offer important external validation of GLP-1RA effects in naturalistic settings and warrant further prospective investigation (Lähteenvuo et al., 2025).

Preclinical studies also exhibited considerable heterogeneity in experimental design, including variation in animal species, dosage regimens, behavioral tasks, and outcome metrics. Many failed to report group-level data in a structured format. Evidence from deprivation and reinstatement paradigms suggests that GLP-1 receptor activation may reduce relapse-like alcohol-seeking following abstinence. This highlights the potential role of GLP-1 signaling in relapse prevention and underscores the need for systematic evaluation of this mechanism in long-term clinical studies (Thomsen et al., 2017). Beyond methodological variability, mechanistic questions also remain unresolved. Animal data suggest that GLP-1 receptor activation may modulate stress-related relapse mechanisms, potentially extending beyond dopaminergic reward pathways. This highlights the need for future studies to clarify how GLP-1R-mediated signaling interacts with stress and neuroendocrine circuits implicated in relapse vulnerability (Schmidt et al., 2016).

### 4.3 Limitations and future directions

Several limitations should be acknowledged. A practical constraint of this review was the difficulty in obtaining complete data from certain clinical studies. In some cases, the author’s contact information was outdated due to institutional changes, which limited access to supplementary or unpublished data. These challenges reflect broader practical barriers to comprehensive data retrieval rather than methodological shortcomings of individual trials. Furthermore, while the included clinical studies employed therapeutic doses consistent with their approved metabolic indications, the heterogeneity in dosing schedules, treatment durations, and outcome measures limits direct comparability across trials. Dose–response relationships and optimal dosing regimens for addiction treatment, therefore, remain to be systematically investigated in future research.

To overcome the identified limitations and advance the field of GLP-1RA research in SUDs, several priority areas must be addressed in future work. First, more large-scale, methodologically rigorous clinical trials are needed across a broader spectrum of SUDs. Expanding research to include opioids and stimulants is particularly urgent, given their public health relevance and promising preclinical results. Additionally, a better understanding is needed of how GLP-1RAs perform across different levels of SUD severity, such as in individuals with treatment-refractory patterns of use, where responses may differ (Bormann et al., 2025).

Second, greater methodological standardization across studies is essential. Variability in outcome measures, sample characteristics, intervention protocols, and follow-up periods contributes to statistical heterogeneity and reduces the interpretability of results. The use of standardized instruments and harmonized study designs would enhance comparability and facilitate meaningful quantitative syntheses.

Third, the potential of combination therapies should be explored systematically. A preclinical study has emphasized the role of neuroinflammation in addiction and suggested that GLP-1RAs, which modulate inflammatory pathways, may enhance the efficacy of existing treatments (Zhu et al., 2022). For instance, pairing GLP-1RAs with buprenorphine (for opioid use disorder) or varenicline (for smoking cessation) could yield synergistic effects, though this hypothesis requires empirical validation through clinical trials.

Fourth, future studies should systematically examine sex-specific treatment responses. Preliminary findings from preclinical studies indicate that biological sex may moderate the efficacy of GLP-1Ras (Herman et al., 2023). Incorporating sex as a core variable in both preclinical and clinical study designs will enhance translational validity and support the development of individualized treatment strategies.

Finally, to mitigate publication bias, it is essential that research includes studies regardless of their outcome significance. This can be achieved through preregistration, open-access repositories, and editorial policies that encourage the publication of inconclusive or null findings.

### 4.4 Conclusion

In conclusion, this systematic review provides a comprehensive and critically informed synthesis of current evidence on the use of GLP-1 receptor agonists in the treatment of SUDs. Preclinical studies provide strong support for the efficacy of GLP-1RA across multiple substances and demonstrate a clear neurobiological rationale. Clinical findings, while preliminary, suggest potential benefits, particularly in alcohol and nicotine use disorders, though current evidence is limited by small sample sizes, short treatment durations, and methodological heterogeneity. The substantial variability in effect estimates observed across studies highlights the need for substance-specific and methodologically harmonized future investigations.

## Data Availability

The original contributions presented in the study are included in the article/Supplementary Material, further inquiries can be directed to the corresponding author.
