# Supplementary material for: The potential role of GLP-1 receptor agonists in substance use disorders – a systematic review
Source: Front Pharmacol. 2026 Jan 2;16:1702448. doi: 10.3389/fphar.2025.1702448 (PMC12808432; doi:10.3389/fphar.2025.1702448)
Supplement: Supplementary file 1 [file DataSheet1.pdf]

Supplementary Material

The Potential Role of GLP-1 Receptor Agonists in Substance Use Disorders – A Systematic Review

Völker M. Kim[1,2], Prechtl Bastian[3], Bormann L. Nicholas[4], Choi Doo-Sup[2,4]

[1]Paracelsus Medical University, Professor-Ernst-Nathan-Straße 1, 90419 Nuremberg, Germany, [2]Department of Molecular Pharmacology and Experimental Therapeutics Mayo Clinic College of Medicine, Rochester, MN, 55905, USA, [3]Klinikum Nürnberg, Breslauer Straße 201, 90471 Nuremberg, Germany, [4]Department of Psychiatry and Psychology, Mayo Clinic College of Medicine, Rochester, MN, 55905, USA

Correspondence and requests for materials should be addressed to Doo-Sup Choi, Ph.D., Department of Molecular Pharmacology and Experimental Therapeutics, Mayo Clinic College of Medicine, 200 First Street SW, Rochester, Minnesota 55905, USA

Phone: (507) 284-5602 Fax: (507) 284-1767 Email: choids@mayo.edu

Supplementary Material 1. Overview of clinically approved GLP-1 receptor agonists and their pharmacological characteristics. The table summarizes the structural properties, administration routes, dosing schedules, and approved therapeutic indications of currently available GLP-1 receptor agonists. These pharmacological features provide essential context for interpreting their potential relevance in addiction-related research.

GLP-1 receptor agonist	Structural class / modification	Route of administration	Dosing frequency	Approved indications	Key pharmacological properties
**Exenatide**	Synthetic exendin-4 analogue (39 amino acids)	Subcutaneous	Twice daily (Byetta®) or once weekly (Bydureon®)	Type 2 diabetes mellitus	Short-acting peptide; resistant to DPP-4 degradation
**Liraglutide**	Acylated human GLP-1 analogue (97% homology)	Subcutaneous	Once daily	Type 2 diabetes mellitus, obesity	Long-acting; binds to albumin via fatty acid chain; central appetite suppression
**Dulaglutide**	GLP-1 fusion protein linked to IgG4 Fc fragment	Subcutaneous	Once weekly	Type 2 diabetes mellitus	High molecular stability; prolonged half-life due to Fc fusion
**Semaglutide**	Acylated GLP-1 analogue with C18 fatty diacid chain	Subcutaneous / oral	Once weekly (s.c.), once daily (oral)	Type 2 diabetes mellitus, obesity	High albumin affinity; long plasma half-life; first oral GLP-1RA formulation
**Lixisenatide**	Synthetic exendin-based peptide (44 amino acids)	Subcutaneous	Once daily	Type 2 diabetes mellitus	Short-acting; enhances postprandial insulin secretion
**Albiglutide**	GLP-1 dimer fused to human albumin	Subcutaneous	Once weekly	Type 2 diabetes mellitus	

Supplementary Material 2. PRISMA 2020 checklist mapping each reporting item to the corresponding section of the manuscript.

PRISMA Item	Description	Location in Manuscript
1. Title	Identified as a systematic review	Title page
2. Abstract	Structured summary provided, including objectives, methods, results, and conclusions.	Abstract
3. Rationale	Explained in the Introduction why this review was necessary.	Introduction, p. 2–3
4. Objectives	Explicit statement of the objectives and research questions.	Introduction, end of p. 3
5. Eligibility criteria	Specified inclusion and exclusion criteria for studies.	Methods, Section 2.2
6. Information sources	Detailed sources: PubMed, Scopus, APA PsycInfo, Embase, Cochrane.	Methods, Section 2.3
7. Search strategy	Full search strategy provided in methods and appendix.	Methods, Section 2.3; Appendix
8. Selection process	Described in detail including reviewer roles and software used.	Methods, Section 2.4
9. Data collection process	Described how data were extracted, who extracted them, and how discrepancies were resolved.	Methods, Section 2.5
10. Data items	All outcomes and study characteristics were clearly defined.	Methods, Section 2.6
11. Study risk of bias assessment	Used Cochrane RoB 2.0 tool for clinical studies.	Methods, Section 2.7; Results 3.3.4
12. Effect measures	Not applicable – narrative synthesis	Methods, Section 2.8
13. Synthesis methods	Narrative synthesis only	Methods, Section 2.9; Results 3.3.3
14. Reporting bias assessment	Publication bias discussed; no formal assessment due to small number of studies.	Discussion, Section 4.1
15. Certainty assessment	Narratiive Review	Methods, Section 2.7; Results 3.3.5
16. Study selection	PRISMA flowchart shows full selection process.	Results, Figure 2
17. Study characteristics	Presented in both text and summary tables.	Results, Section 3.1 and Tables 1–2
18. Risk of bias in studies	RoB 2.0 assessments detailed in methods and results.	Methods 2.7; Results 3.3.4
19. Results of individual studies	Summarized and presented in text	Results, Section 3.3.3.2
20. Results of syntheses	Narrative	Results, Sections 3.3.3.2–3.3.3.4
21. Reporting biases	Narrative	Results 3.3.4; Figure 5
22. Certainty of evidence	Narrative	Results, Section 3.3.5
23. Discussion	Interpretation, limitations, implications and future directions discussed.	Discussion, Section 4

24. Registration and protocol	Registered in PROSPERO: CRD42024571356.	Methods, Section 2.1
25. Support	Acknowledgements section describes support received.	Acknowledgements
26. Competing interests	Stated in the manuscript that there are none.	Conflict of Interest Statement
27. Availability of data, code and other materials	Data and methods described; software tools specified.	Methods, Sections 2.8–2.9

Supplementary Material 3. Full electronic search strategies were used for the systematic literature search across PubMed, Scopus, APA PsycInfo, Embase, and the Cochrane Central Register of Controlled Trials (conducted August 8, 2024). Search terms included Medical Subject Headings (MeSH) and free-text terms related to glucagon-like peptide-1 receptor agonists (GLP-1RAs) and substance use disorders (SUDs). Filters were applied for publication date (2014–2024) and language (English or German).

Full Search Strategies

GLP-1 and substance use disorders

Database Search: conducted August 8, 2024

PubMed (pubmed.ncbi.nlm.nih.gov) search strategy

#	Search Query	Results
1	"glucagon like peptide 1"[Title/Abstract] OR "GLP 1"[Title/Abstract] OR "GLP1"[Title/Abstract] OR "incretin mimetics"[Title/Abstract] OR "semaglutide*"[Title/Abstract] OR "liraglutide*"[Title/Abstract] OR "tirzepatide*"[Title/Abstract] OR "lixisenatide*"[Title/Abstract] OR "liraglutide*"[Title/Abstract] OR "exenatide*"[Title/Abstract] OR "dulaglutide*"[Title/Abstract]	26,006
2	"Glucagon-Like Peptide-1 Receptor Agonists"[Mesh]	267
3	1 OR 2	26,016
4	"substance-related disorder*"[Title/Abstract] OR "substance use*"[Title/Abstract] OR "substance abuse*"[Title/Abstract] OR "addictive disorder*"[Title/Abstract] OR "drug use disorder*"[Title/Abstract] OR "drug habituation*"[Title/Abstract] OR "substance dependence*"[Title/Abstract] OR "chemical dependence*"[Title/Abstract] OR "substance addict*"[Title/Abstract] OR "drug dependence*"[Title/Abstract] OR "drug addict*"[Title/Abstract] OR "drug abuse*"[Title/Abstract] OR "drug misuse*"[Title/Abstract] OR alcohol*[Title/Abstract] OR cocaine*[Title/Abstract] OR heroine*[Title/Abstract] OR "amphetamine related disorder*"[Title/Abstract] OR "amphetamine addict*"[Title/Abstract] OR "amphetamine abuse*"[Title/Abstract] OR "tobacco use disorder*"[Title/Abstract] OR "nicotine dependence*"[Title/Abstract] OR "tobacco dependence*"[Title/Abstract] OR "nicotine addict*"[Title/Abstract] OR "narcotic related disorder*"[Title/Abstract] OR "narcotic abuse*"[Title/Abstract] OR "narcotic dependence*"[Title/Abstract] OR "narcotic addict*"[Title/Abstract] OR addiction[Title/Abstract] OR "addictive behavior*"[Title/Abstract]	582,251
5	"Substance-Related Disorders"[Mesh] OR "Alcohol-Related Disorders"[Mesh] OR "Cocaine-Related Disorders"[Mesh] OR "Drug Misuse"[Mesh] OR "Amphetamine-Related Disorders"[Mesh] OR "Tobacco Use Disorder"[Mesh] OR "Narcotic-Related Disorders"[Mesh] OR "Behavior, Addictive"[Mesh]	329,595
6	4 OR 5	713,160
7	3 AND 6	762
8	7 AND (2014/01/01:3000/12/31[Date - Publication]) AND (English[Filter] OR German[Filter])	659

Scopus (scopus.com)

#	Search Query	Results

1	TITLE-ABS("glucagon like peptide 1" OR "GLP 1" OR "GLP1" OR "incretin mimetics" OR semaglutide* OR liraglutide* OR tirzepatide* OR lixisenatide* OR liraglutide* OR exenatide* OR dulaglutide*)	28,131
2	TITLE-ABS("substance-related disorder*" OR "substance use*" OR "substance abuse*" OR "addictive disorder*" OR "drug use disorder*" OR "drug habituation*" OR "substance dependence*" OR "chemical dependence*" OR "substance addict*" OR "drug dependence*" OR "drug addict*" OR "drug abuse*" OR "drug misuse*" OR alcohol* OR cocaine* OR heroine* OR "amphetamine related disorder*" OR "amphetamine addict*" OR "amphetamine abuse*" OR "tobacco use disorder*" OR "nicotine dependence*" OR "tobacco dependence*" OR "nicotine addict*" OR "narcotic related disorder*" OR "narcotic abuse*" OR "narcotic dependence*" OR "narcotic addict*" OR addiction OR "addictive behavior*")	973,191
3	1 AND 2	714
4	3 AND PUBYEAR > 2013 AND (LIMIT-TO (LANGUAGE, "German") OR LIMIT-TO (LANGUAGE, "English"))	579

APA PsycInfo (Ovid platform, 1967 to July Week 5 2024) search strategy

#	Search Query	Results
1	("glucagon like peptide 1" or "GLP 1" or "GLP1" or "incretin mimetics" or semaglutide* or liraglutide* or tirzepatide* or lixisenatide* or liraglutide* or exenatide* or dulaglutide*).ti. or ("glucagon like peptide 1" or "GLP 1" or "GLP1" or "incretin mimetics" or semaglutide* or liraglutide* or tirzepatide* or lixisenatide* or liraglutide* or exenatide* or dulaglutide*).ab.	875
2	"Glucagon-Like Peptide-1 Receptor Agonists"/	0
3	1 or 2	875
4	("substance-related disorder*" or "substance use*" or "substance abuse*" or "addictive disorder*" or "drug use disorder*" or "drug habituation*" or "substance dependence*" or "chemical dependence*" or "substance addict*" or "drug dependence*" or "drug addict*" or "drug abuse*" or "drug misuse*" or alcohol* or cocaine* or heroine* or "amphetamine related disorder*" or "amphetamine addict*" or "amphetamine abuse*" or "tobacco use disorder*" or "nicotine dependence*" or "tobacco dependence*" or "nicotine addict*" or "narcotic related disorder*" or "narcotic abuse*" or "narcotic dependence*" or "narcotic addict*" or addiction or "addictive behavior*").ti. or ("substance-related disorder*" or "substance use*" or "substance abuse*" or "addictive disorder*" or "drug use disorder*" or "drug habituation*" or "substance dependence*" or "chemical dependence*" or "substance addict*" or "drug dependence*" or "drug addict*" or "drug abuse*" or "drug misuse*" or alcohol* or cocaine* or heroine* or "amphetamine related disorder*" or "amphetamine addict*" or "amphetamine abuse*" or "tobacco use disorder*" or "nicotine dependence*" or "tobacco dependence*" or "nicotine addict*" or "narcotic related disorder*" or "narcotic abuse*" or "narcotic dependence*" or "narcotic addict*" or addiction or "addictive behavior*").ab.	257,647
5	"Substance-Related Disorders"/ OR "Alcohol-Related Disorders"/ OR "Cocaine-Related Disorders"/ OR "Drug Misuse"/ OR "Amphetamine-Related Disorders"/ OR "Tobacco Use Disorder"/ OR "Narcotic-Related Disorders"/ OR "Behavior, Addictive"/	345
6	4 or 5	257,764

7	3 and 6	73
8	limit 7 to ((english or german) and yr="2014 -Current")	62

EMBASE (Ovid platform, 1974 to 2024 August 07) search strategy

#	Search Query	Results
1	("glucagon like peptide 1" or "GLP 1" or "GLP1" or "incretin mimetics" or semaglutide* or liraglutide* or tirzepatide* or lixisenatide* or liraglutide* or exenatide* or dulaglutide*).ti. or ("glucagon like peptide 1" or "GLP 1" or "GLP1" or "incretin mimetics" or semaglutide* or liraglutide* or tirzepatide* or lixisenatide* or liraglutide* or exenatide* or dulaglutide*).ab.	41,706
2	"Glucagon-Like Peptide-1 Receptor Agonists"/	12,831
3	1 or 2	46,538
4	("substance-related disorder*" or "substance use*" or "substance abuse*" or "addictive disorder*" or "drug use disorder*" or "drug habituation*" or "substance dependence*" or "chemical dependence*" or "substance addict*" or "drug dependence*" or "drug addict*" or "drug abuse*" or "drug misuse*" or alcohol* or cocaine* or heroine* or "amphetamine related disorder*" or "amphetamine addict*" or "amphetamine abuse*" or "tobacco use disorder*" or "nicotine dependence*" or "tobacco dependence*" or "nicotine addict*" or "narcotic related disorder*" or "narcotic abuse*" or "narcotic dependence*" or "narcotic addict*" or addiction or "addictive behavior*").ti. or ("substance-related disorder*" or "substance use*" or "substance abuse*" or "addictive disorder*" or "drug use disorder*" or "drug habituation*" or "substance dependence*" or "chemical dependence*" or "substance addict*" or "drug dependence*" or "drug addict*" or "drug abuse*" or "drug misuse*" or alcohol* or cocaine* or heroine* or "amphetamine related disorder*" or "amphetamine addict*" or "amphetamine abuse*" or "tobacco use disorder*" or "nicotine dependence*" or "tobacco dependence*" or "nicotine addict*" or "narcotic related disorder*" or "narcotic abuse*" or "narcotic dependence*" or "narcotic addict*" or addiction or "addictive behavior*").ab.	765,631
5	"Substance-Related Disorders"/ OR "Alcohol-Related Disorders"/ OR "Cocaine-Related Disorders"/ OR "Drug Misuse"/ OR "Amphetamine-Related Disorders"/ OR "Tobacco Use Disorder"/ OR "Narcotic-Related Disorders"/ OR "Behavior, Addictive"/	220,412
6	4 or 5	863,160
7	3 and 6	1,535
8	limit 7 to ((english or german) and yr="2014 -Current")	1,319

Cochrane Central Register of Controlled Trials (Ovid platform, EBM Reviews, July 2024) search strategy

#	Search Query	Results
1	("glucagon like peptide 1" or "GLP 1" or "GLP1" or "incretin mimetics" or semaglutide* or liraglutide* or tirzepatide* or lixisenatide* or liraglutide* or exenatide* or dulaglutide*).ti. or ("glucagon like peptide 1" or "GLP 1" or "GLP1" or "incretin mimetics" or semaglutide* or liraglutide* or tirzepatide* or lixisenatide* or liraglutide* or exenatide* or dulaglutide*).ab.	9,382
2	"Glucagon-Like Peptide-1 Receptor Agonists"/	19
3	1 or 2	9,383

4	("substance-related disorder*" or "substance use*" or "substance abuse*" or "addictive disorder*" or "drug use disorder*" or "drug habituation*" or "substance dependence*" or "chemical dependence*" or "substance addict*" or "drug dependence*" or "drug addict*" or "drug abuse*" or "drug misuse*" or alcohol* or cocaine* or heroine* or "amphetamine related disorder*" or "amphetamine addict*" or "amphetamine abuse*" or "tobacco use disorder*" or "nicotine dependence*" or "tobacco dependence*" or "nicotine addict*" or "narcotic related disorder*" or "narcotic abuse*" or "narcotic dependence*" or "narcotic addict*" or addiction or "addictive behavior*").ti. or ("substance-related disorder*" or "substance use*" or "substance abuse*" or "addictive disorder*" or "drug use disorder*" or "drug habituation*" or "substance dependence*" or "chemical dependence*" or "substance addict*" or "drug dependence*" or "drug addict*" or "drug abuse*" or "drug misuse*" or alcohol* or cocaine* or heroine* or "amphetamine related disorder*" or "amphetamine addict*" or "amphetamine abuse*" or "tobacco use disorder*" or "nicotine dependence*" or "tobacco dependence*" or "nicotine addict*" or "narcotic related disorder*" or "narcotic abuse*" or "narcotic dependence*" or "narcotic addict*" or addiction or "addictive behavior*").ab.	52,387
5	"Substance-Related Disorders"/ OR "Alcohol-Related Disorders"/ OR "Cocaine-Related Disorders"/ OR "Drug Misuse"/ OR "Amphetamine-Related Disorders"/ OR "Tobacco Use Disorder"/ OR "Narcotic-Related Disorders"/ OR "Behavior, Addictive"/	10,142
6	4 or 5	55,263
7	3 and 6	285
8	limit 7 to ((english or german) and yr="2014 -Current")	250

Studies Identified – 2,869; Duplicates removed -1,324; Studies screened – 1,545; Full-text studies assessed – 218; Studies included – 45

Supplementary Material 4. Characteristics of included preclinical and clinical studies investigating GLP-1 receptor agonists (GLP-1RAs) in substance use disorders (SUDs). Data include study type, substance investigated, population/sample, intervention, control condition, treatment duration, and outcomes. AUD = alcohol use disorder; CUD = cocaine use disorder; OUD = opioid use disorder; NAc = nucleus accumbens; VTA = ventral tegmental area; NTS = nucleus tractus solitarius; IVSA = intravenous self-administration; CPP = conditioned place preference.

Study	Type	Substance	Population	GLP-1RA	Control	Duration	Outcomes
Klausen et al. (2022)	Clinical (RCT)	Alcohol	127 patients with AUD (DSM-5 criteria), mean age 52, 60% male	Exenatide (2 mg s.c. weekly)	Placebo + standard CBT	26 weeks + 6-month follow-up	Primary: Reduction in heavy drinking days (TLFB); Secondary: fMRI cue-reactivity, SPECT DAT availability, alcohol intake, craving, BMI, HbA1c
Probst et al. (2023)	Clinical (Secondary)	Alcohol (in smokers)	151 alcohol-consuming participants out of 255 enrolled smokers, median age 42, 61% female; high BMI prevalence (>90%)	Dulaglutide (0.75 mg then 1.5 mg weekly s.c.)	Placebo + standard smoking	12 weeks	Primary: Weekly alcohol consumption (standard glasses/week); Secondary: smoking abstinence, new drinking onset, drug use, interaction with smoking
Aranäs et al. (2023, eBioMedicine)	Preclinical (Rodent	Alcohol	Male and female Wistar rats and NMRI mice; intermittent access alcohol model; n varies (e.g., n=24 per group); both sexes used	Semaglutide (0.026–0.052 mg/kg, s.c.)	Vehicle	Multiple acute and repeated treatments (up to 5 sessions); relapse model, CPP, and dopamine experiments over several weeks	Reduced alcohol intake and relapse-like drinking; attenuated alcohol-induced locomotor activity and dopamine release in nucleus accumbens; increased dopamine release
Vallöf et al. (2015)	Preclinical (Rodent	Alcohol	Mice (CPP, dopamine microdialysis) and Wistar rats (intermittent access, alcohol deprivation, operant self-administration)	Liraglutide (0.05–0.1 mg/kg s.c.)	Vehicle	Acute and repeated administration (1–8 days), across several behavioral paradigms	Attenuated alcohol-induced dopamine release in nucleus accumbens and conditioned place preference; reduced alcohol intake, relapse-like drinking, operant self-administration
Herman et al. (2023)	Preclinical (Rodent	Nicotine	Male and female Sprague-Dawley rats (n=121); self-administration and withdrawal models; yoked-saline controls; both sexes equally	Liraglutide (25 µg/kg i.p.)	Vehicle	Acute: single dose prior to final nicotine self-administration; Chronic: 10-day abstinence phase with daily dosing	Reduced nicotine self-administration and reinstatement; attenuated withdrawal-induced hyperphagia and body weight gain; no effect on water intake
Douton et al. (2021)	Preclinical (Rodent	Opioids (Heroin)	Male Sprague-Dawley rats (n=55); heroin self-administration, cue-induced and drug-induced reinstatement models	Exendin-4 (2.4 µg/kg i.p.)	Vehicle	15-day heroin self-administration; 16-day abstinence with daily treatment; reinstatement tests over 10+ days	Ex-4 reduced cue-induced and drug-induced heroin seeking (only with 1h pretreatment); increased saccharin acceptance in vulnerable rats; elevated OX1
Hernandez et al. (2019)	Preclinical (Rodent	Cocaine	Male Sprague-Dawley rats; self-administration, extinction, reinstatement models; cocaine-experienced and yoked saline	Exendin-4 (0.005–0.05 µg intra-NAc; 0.1–0.2 µg/kg	Vehicle (aCSF)	21 days cocaine SA, extinction up to 7 days; single or repeated Ex-4 administration prior to reinstatement tests	Systemic and intra-nucleus accumbens Ex-4 attenuated cocaine-primed reinstatement; increased MSN firing (but not synaptic transmission); no effect on sucrose
Schmidt et al. (2016)	Preclinical (Rodent	Cocaine	Male Sprague-Dawley rats; cocaine self-administration, progressive ratio (PR), AAV-shRNA GLP-1R knockdown, VTA microinjections	Exendin-4 (0.005–0.05 µg intra-VTA); Exendin-(9–39)	Vehicle	21 days cocaine SA; Ex-4 administered acutely; corticosterone via 4th ventricle; GLP-1R knockdown assessed over 14+ days	Intra-VTA Ex-4 reduced cocaine but not sucrose SA; corticosterone increased NTS GLP-1 activation and suppressed cocaine SA; Ex-4 antagonist blocked
Díaz-Megido et al. (2023)	Preclinical (Rodent	Alcohol	Male and female C57BL/6J mice; operant oral alcohol self-administration and cue-induced reinstatement; n = 34M / 39F	Exendin-4 (1.8 and 3.2 µg/kg i.p.)	Vehicle (saline)	Acute: single injection 30 min prior to reinstatement or self-administration testing	In males: both doses reduced alcohol self-administration and completely suppressed reinstatement of alcohol seeking; in females
Vallöf et al. (2020)	Preclinical (Rodent	Alcohol	Male and female outbred Rcc Han Wistar rats (n ≥ 10/group); intermittent access 20% ethanol two-bottle choice	Dulaglutide (0.05 or 0.1 mg/kg, s.c., once weekly)	Vehicle	5 or 9 weeks treatment + up to 6 weeks follow-up (no treatment); total: 8–15 weeks	Reduced ethanol intake and preference in both sexes; effects more persistent in males after discontinuation; sex-specific differences in monoamine signaling
Abtahi et al. (2018)	Preclinical (Rodent	Alcohol	Female Sprague-Dawley rats (n=19); 12-week intermittent 2-bottle choice alcohol exposure; microinjections into NAcC or NAcS	Exendin-4 (0.025–0.05 µg into NAcC/NAcS)	Vehicle; ghrelin antagonist	Acute: behavioral assessment at 2, 6, 24 h post-injection	Ex-4 in NAcS, but not NAcC, reduced alcohol intake; combination with ghrelin antagonist enhanced effect; no estrous effect on alcohol;

Aranäs et al. (2023, Frontiers)	Preclinical (Rodent	Alcohol	Male and female Rcc/Han Wistar rats; intermittent alcohol access paradigm; n=8–24 per group across 3 experiments	Semaglutide (0.026 mg/kg, s.c.), in combination with	Vehicle, mono-therapies	10-week alcohol baseline, followed by 4 sessions of acute/repeated drug exposure; HFD exposure over 3 weeks; follow-up injections post-	Semaglutide reduced alcohol intake and preference in both sexes; no additive effects with varenicline, bupropion, or HFD
Thomsen et al. (2017)	Preclinical (Rodent	Alcohol	Male C57BL/6NTac mice (n = 15), group-housed; continuous alcohol access for 37 days, followed by 10-day deprivation and re-exposure	Exendin-4 (1.5 µg/kg/day , s.c.)	Saline	8 days of treatment during deprivation, followed by 8 days post-reintroduction of alcohol, and 7-day washout period	Exendin-4 prevented alcohol deprivation-induced increases in intake and preference; prolonged latency to first drink; fewer drinking bouts
Sørensen et al. (2016)	Preclinical (Rodent	Alcohol	Male C57BL/6J mice (n = 14); intravenous ethanol self-administration (IVSA); reinforcement criteria ≥15 infusions/session for ≥3	Exendin-4 (3.2 µg/kg i.p.)	Saline	14–18 daily 2h IVSA sessions until stable intake; acute Ex-4 testing with 2 baseline sessions between doses	Exendin-4 decreased IV ethanol self-administration by ≥70%; no significant effect on operant responding for palatable food; effect interpreted as specific to GLP-1 Agonist
Dixon et al. (2020)	Preclinical (Rodent)	Alcohol	Male Long-Evans rats (n = 22); intermittent access to 20% alcohol; trained for operant alcohol self-administration and reacquisition	Exendin-4 (0.01 or 0.05 µg, intra-VTA)	Saline (vehicle); within-subject	Operant alcohol self-administration for 4 weeks (FR1); followed by reacquisition, progressive ratio (PR), and locomotor testing	Exendin-4 in VTA reduced alcohol self-administration in high alcohol drinkers (HAD); no effect on reacquisition, PR performance, or locomotion
Allingbjerg et al. (2022)	Preclinical (Rodent)	Alcohol	Male C57BL/6NTac mice (n = 31); oral alcohol self-administration in operant conditioning paradigm with implanted cannulas targeting	Exendin-4 (3.2–32 ng/hemisphere)	Vehicle (aCSF) infusion	Baseline self-administration sessions for stabilization, followed by randomized microinfusions and testing with 30-min pretreatment time	Exendin-4 in lateral septum (LS), ventral hippocampus, and nucleus accumbens (NAc) significantly reduced alcohol self-administration
Vallöf et al. (2019)	Preclinical (Rodent)	Alcohol	Male NMRI mice (CPP, locomotor activity, microdialysis) and male outbred Wistar rats (intermittent access); total duration up to 12 weeks	Exendin-4 (0.025–0.05 µg/site, intra-NTS)	Vehicle (Ringer solution or NaCl)	Various acute and chronic paradigms: single Ex4 administration in mice; 12-week intermittent access with alcohol in rats	Intra-NTS Ex4 reduced alcohol-induced locomotor stimulation, accumbal dopamine release, and conditioned place preference in mice
Chuong et al. (2023)	Preclinical (Rodent)	Alcohol	Male and female C57BL/6J mice (binge-like drinking; n=77), male and female Wistar rats (binge-like and dependence-induced drinking;	Semaglutide (0.001–0.1 mg/kg, s.c.)	Vehicle	Multiple sessions over weeks for drinking-in-the-dark in mice; chronic intermittent alcohol vapor exposure for dependence in rats	Semaglutide reduced binge-like and dependence-induced alcohol intake in both
Marty et al. (2020)	Preclinical (Rodent)	Alcohol	Male Wistar rats (n=24 across two cohorts); intermittent access 2-bottle choice paradigm (10% ethanol)	Liraglutide (0.1 mg/kg, i.p.) and Semaglutide (0.1	Vehicle	Acute injections after stable alcohol intake; outcomes assessed on day of injection and up to 12 days post-injection	Liraglutide and semaglutide acutely reduced ethanol intake and body weight. Semaglutide also reduced ethanol preference. Effects were transient.
Fink-Jensen et al. (2024)	Preclinical (primate)	Alcohol	20 male alcohol-preferring vervet monkeys; tested with 10% ethanol (4 h/day access), aged young adults (4.4–6.5 kg)	Semaglutide (up to 0.05 mg/kg, s.c., 2x/week)	Vehicle (saline solution)	10 days baseline, 3 weeks up-titration without alcohol, followed by 3 weeks treatment + alcohol access and 1 week washout (total ~7 weeks)	Semaglutide significantly reduced alcohol consumption during weeks 1 and 2 (trend in week 3); no effect during washout; no emesis or weight change
Vallöf et al. (2019)	Preclinical	Alcohol	Adult male NMRI mice and male Wistar rats (post-pubertal), exposed to 12-week intermittent access to 20% alcohol (rats), or 1.75 g/kg	Exendin-4 (Ex4), microinjected bilaterally	Vehicle (Ringer solution)	Mice: acute tests (locomotor activity, CPP); Rats: 12 weeks intermittent access followed by Ex4 microinjection	Ex4 into NAc shell and LDTg blocked alcohol-induced locomotor stimulation in mice and reduced alcohol intake in high-drinking rats. No effect in aVTA or pVTA
Colvin et al. (2020)	Preclinical	Alcohol	Adult male Sprague Dawley rats, tested via two-bottle choice paradigm for alcohol and operant conditioning for sucrose pellets	Exendin-4 (Ex-4), injected into brain regions: VTA,	Vehicle (sterile isotonic saline)	Operant conditioning over 2 weeks training; alcohol testing during dark cycle after 12-week	Ex-4 in VTA, NAcC, NAcS, LH, and DMHipp significantly reduced alcohol consumption; ArcN and PVN had no alcohol effect but suppressed sucrose

						intermittent access to alcohol	
Colvin et al. (2022)	Preclinical	Alcohol + Cocaine /Amphetamine	Adult male Sprague-Dawley rats (N=80); tested in various groups with systemic and intra-VTA injections of GLP-1 agonist Exendin-4, ghrelin,	Exendin-4 (Ex-4), intra-VTA injections at 0.01, 0.05,	Vehicle (saline)	Habituation with 6% ethanol across multiple sessions;	Ex-4 reduced ethanol intake and reversed the stimulatory effects of D-amphetamine and cocaine on ethanol consumption; Ex-4 blocked ghrelin
Sirohi et al. (2016)	Preclinical	Alcohol, Amphetamine	GLP-1R KDNestin mice (central GLP-1R knockout) and wild-type FLOX mice (n = 13/group)	Exendin-4 (30 µg/kg, i.p.)	Saline	Acute testing for amphetamine-CPP, 90-minute alcohol intake, and 4-hour hedonic feeding paradigm following 21h deprivation	EX-4 blocked amphetamine CPP and alcohol intake in FLOX mice but not in GLP-1R KDNestin mice, indicating central GLP-1R mediation
Thomsen et al. (2019)	Preclinical (Non-human	Alcohol	Male African vervet monkeys (Cercopithecus aethiops); n = 32; alcohol-preferring subpopulation selected after baseline screening	Exenatide (0.04 mg/kg, weekly, 2 weeks);	Vehicle (Bydureon®)	Exenatide: 5-week up-titration without alcohol, followed by 2-week treatment with alcohol access and 1-week washout; Liraglutide: 2-week	Both GLP-1RAs significantly reduced alcohol intake, with stronger effects for liraglutide. Reductions were evident during treatment weeks.
Brigande et al. (2023)	Preclinical	Alcohol	Adult male Sprague Dawley rats (N=48); bilateral cannulae targeting the VTA; tested for operant sucrose responding and ethanol intake	Exendin-4 (0.01–0.1 µg, intra-VTA)	Vehicle (saline); neuropeptide Y	Operant training; repeated measures for Ex-4 paired with NPY or ghrelin; ethanol access after stabilization with 6% solution	NPY and ghrelin increased sucrose responding and ethanol intake; Ex-4 dose-dependently blocked these effects.
Lüthi et al. (2024)	Clinical (RCT)	Nicotine	296 adult smokers enrolled in a double-blind, placebo-controlled RCT; assessed for smoking cessation and weight management	Dulaglutide (1.5 mg/week, subcutaneous)	Placebo	12-week intervention + 52-week total follow-up (longitudinal post-treatment analysis)	Primary: Smoking abstinence at weeks 12, 24, and 52 (biochemically verified) Secondary: weight gain prevention, craving, mood. No significant differences in
Yammine et al. (2021)	Clinical (Pilot RCT)	Nicotine	84 treatment-seeking, prediabetic and/or overweight adult smokers; 82 completed modified ITT sample	Exenatide ER (2 mg, once weekly, subcutaneous)	Placebo + Nicotine	6-week treatment with 7-day point-prevalence abstinence, craving, withdrawal, and post-cessation weight gain measured	Exenatide increased abstinence rates (46.3% vs. 26.8%; PP = 96.5%), reduced craving and withdrawal symptoms among abstainers, and mitigated weight gain
Angarita et al. (2021)	Clinical (Human)	Cocaine	13 non-treatment-seeking adults with moderate to severe cocaine use disorder (DSM-5); mostly male and African American;	Exenatide (5 mcg, subcutaneous, single dose)	Placebo (saline)	Single-day crossover sessions; participants received exenatide or placebo 3 hours prior to controlled cocaine self-administration	No significant differences in cocaine self-administration, subjective euphoria, or craving between conditions
Tuesta et al. (2017)	Preclinical (Rodent)	Nicotine	Male and female C57BL/6 mice; wild-type and global GLP-1R knockout; self-administration and reinstatement models	Liraglutide (200 µg/kg, i.p.)	Saline; GLP-1R KO mice	10-day self-administration; extinction and reinstatement testing; acute liraglutide prior to reinstatement	Liraglutide attenuated cue- and stress-induced reinstatement in wild-type but not GLP-1R KO mice; KO mice showed increased nicotine intake and seeking
Hernandez et al. (2021)	Preclinical (Rodent)	Cocaine	Male Sprague-Dawley rats; cocaine IV self-administration; examined GLP-1 neuron activation and receptor distribution in NTS and VTA	Endogenous GLP-1 activation via cocaine; no	Saline; Yoked saline controls	21 days of cocaine or saline self-administration; sacrifice 24 h after last session for histology/immunofluorescence	Cocaine increased GLP-1 and GLP-1R expression in caudal NTS and VTA; identified anatomical basis for GLP-1 signaling in reward circuits
Hernandez et al. (2018)	Preclinical (Rodent)	Cocaine	Male Sprague-Dawley rats; cocaine self-administration and reinstatement model	Exendin-4 (systemic and intra-VTA administration)	Vehicle; GLP-1R antagonist	21 days of cocaine self-administration, extinction phase, reinstatement tests; multiple reinstatement protocols including cue- and cocaine-	Systemic Exendin-4 dose-dependently attenuated cocaine seeking at sub-threshold doses (0.1–0.2 µg/kg); effects mediated via GLP-1R in the VTA; no effect
Sørensen et al. (2015)	Preclinical	Cocaine	Male NMRI and C57Bl/6 mice; acute and chronic cocaine self-	Exendin-4 (0.3–100	Saline	Acute and chronic self-administration protocols; various	Exendin-4 reduced both acute and chronic cocaine self-administration;

	(Rodent)		administration, locomotor activity, microdialysis, and c-fos	µg/kg i.p.)		doses and administration times; includes 21-day procedures and microdialysis	attenuated cocaine-induced hyperlocomotion
Zhu et al. (2022)	Preclinical (Rodent)	Cocaine	Male and female mice; microglial GLP-1R knockout (Cx3cr1CreER:Glp1rfl/fl) vs. controls; cocaine sensitization,	Endogenous GLP-1 activation; chemogenetic	Wild-type and vehicle-treated	Cocaine injections over 5 days; testing for locomotor sensitization, gene expression (RNA-seq, qPCR)	Microglial GLP-1R deletion enhanced cocaine-induced locomotor sensitization and neuroinflammation; DREADD-induced activation of GLP-1R-expressing
Urbanik et al. (2022)	Preclinical (Rodent)	Opioid (Fentanyl)	Male rats; fentanyl self-administration model; extinction, reinstatement, locomotor activity, weight, and plasma liraglutide	Liraglutide (0.3 mg/kg, s.c.)	Saline;	Extinction and reinstatement paradigm with acute liraglutide or saline administration prior to cue- and drug-induced reinstatement sessions	Liraglutide significantly reduced cue- and drug-induced reinstatement of fentanyl seeking without affecting general locomotion or body weight
Douton et al. (2022)	Preclinical (Rodent)	Alcohol	Male Wistar rats exposed to intermittent access to ethanol; tested for alcohol intake and relapse-like drinking	Semaglutide (0.003–0.03 mg/kg, s.c.)	Saline (vehicle)	Chronic daily treatment for 14 days	Dose-dependent reduction in alcohol intake; suppressed relapse-like drinking behavior after abstinence
Bornebusch et al. (2019)	Preclinical (Rodent)	Opioid (Heroin)	Male Sprague-Dawley rats (n=48); heroin self-administration model	Liraglutide (0.3 mg/kg, s.c.)	Saline (vehicle)	Acute and chronic paradigms (3–7 days)	Significantly reduced heroin-seeking induced by cues, stress, and heroin priming; no effect on locomotor activity or natural reward
Douton et al. (2022)	Preclinical (Rodent	Heroin	Rats trained to self-administer heroin; examined reinstatement models	Liraglutide (0.3 mg/kg, s.c.)	Saline	Acute administration before reinstatement tests	Liraglutide significantly attenuated heroin-seeking behavior triggered by cues, stress, or heroin priming
Suchankova et al. (2015)	Preclinical (Mouse +Human)	Alcohol	GLP-1R knockout and wild-type C57BL/6J mice; additionally, human genetic data from NIAAA sample on GLP1R polymorphisms and	Genetic disruption (KO); no pharmacological	Wild-type controls;	Chronic alcohol vapor exposure, behavioral analysis	GLP-1R KO mice showed increased alcohol intake and preference. Human SNPs in GLP1R were associated with risk for alcohol dependence.
Lengsfeld et al. (2023)	Clinical (RCT)	Nicotine (Smoking	120 adult smokers; randomized, double-blind, placebo-controlled single-site trial assessing dulaglutide in smoking cessation	Dulaglutide (1.5 mg weekly, s.c.)	Placebo	12-week treatment; short-term outcome assessment	No significant difference in smoking abstinence; transient reduction in weight gain during treatment
Evans et al. (2022)	Preclinical (Rodent)	Opioids (Heroin)	Male high drug-taking rats trained on heroin self-administration; dose titration study	Liraglutide (0.1–0.6 mg/kg)	Saline	Acute dose titration and challenge test	Dose-dependent suppression of cue- and drug-induced heroin seeking; doses produced sedation but reduced relapse-like behavior
Hendershot et al. (2025)	Clinical (RCT)	Alcohol	127 adults with DSM-5 alcohol use disorder	Semaglutide 0.25–1.0 mg s.c., once weekly	Placebo	26 weeks	Percent heavy drinking days; drinks per drinking day; craving scores
